# The knowledge level and influencing factors of sarcopenia among Chinese community-dwelling older adults

**DOI:** 10.1371/journal.pone.0333557

**Published:** 2025-10-16

**Authors:** Wanyue Guo, Tianya Wang, Huohuo Dai, Yuqi Wang, Zihan Zhou, Ye Wang, Xianbo Pei, Qing Zhang

**Affiliations:** 1 School of Nursing, Wuhan University, Wuhan, **Hubei Province**, China; 2 Department of Emergency, Renmin Hospital of Wuhan University, Wuhan, Hubei Province, China; 3 Department of Public Health and Primary Care, Leiden University Medical Centre, Leiden, Netherlands; Fondazione Don Carlo Gnocchi, ITALY

## Abstract

**Aim:**

This study aimed to assess the knowledge level of sarcopenia and its influencing factors among Chinese community-dwelling older adults.

**Methods:**

We conducted a cross-sectional study using the sociodemographic questionnaire, the Sarcopenia Knowledge Awareness Survey Scale and the SARC-CalF scales for data collection in 5 communities in Wuhan, Hubei Province, China. Influencing factors of sarcopenia knowledge were analyzed by logistic regression, and a decision tree model.

**Results:**

A total of 569 participants were included in the analysis. Only 17.8% participants had heard of sarcopenia, 19.3% of participants screened positive for sarcopenia, and 29.0% had a self-assessed muscle status that was inconsistent with the actual screened sarcopenia status. Those aged 75 years and above were more likely to have inadequate sarcopenia knowledge (OR=0.468,95% CI = 0.303–0.723). The group with the highest proportion of low sarcopenia knowledge consisted of those who lived alone or with children, had an education level of middle school or below, and never engaged in any social activities (97.6%).

**Conclusions:**

Knowledge of sarcopenia among older adults in Chinese communities is still inadequate. Further tailored interventions may help to ameliorate this lack of knowledge.

## Introduction

Sarcopenia, as one of the geriatric syndromes, is characterized by a loss of muscle mass, accompanied by a decline in muscle strength and or physical function [1]. Depending on different definitions of sarcopenia, its estimated prevalence ranges from 9.9 to 40.4% in the elderly [2]. Sarcopenia is an age-related condition that also causes multiple adverse health outcomes, including decreased self-care ability and quality of life, overall functional decline, and even death [3]. In fact, sarcopenia is a modifiable and dynamic process, that can be effectively avoided and reversed by early identification, screening, and prevention of modifiable risk factors [4].

However, despite its clinical significance, awareness of sarcopenia remains alarmingly low among older populations. Previous studies have shown that the older population frequently have a lack of understanding. This lack of knowledge itself may prevent older adults from accurately assessing their muscle health status, causing them to miss opportunities for early intervention. For instance, Dutch research revealed that most elderly individuals lack comprehension of sarcopenia, with only a minority of affected persons accurately recognize their condition [5,6]. Similarly, a Malaysian study reported public awareness hovers around low-to-moderate levels, strongly influenced by age and education [7]. This pervasive knowledge gap results in missed opportunities for early detection and intervention among at-risk groups.

Because the onset of sarcopenia is insidious and slow, the Asian Sarcopenia Working Group recommends timely identification of high-risk groups for effective intervention [8]. It is therefore crucial to improve awareness of sarcopenia among older adults, enhance their understanding of its risk factors, emphasize preventive approaches, and promote preventive behaviors. These steps may help to mitigate the onset and progression of sarcopenia at its root, ultimately reducing functional decline and healthcare costs [5,9].

Disease awareness is influenced by various factors, including age, education level, occupation, and social environment. Previous studies have shown that a patient’s status of sarcopenia affects their understanding of the condition [10]. If individuals perceive their health as adequate, they may become less motivated to seek medical or social support, which could result in reduced awareness of sarcopenia [11]. Therefore, assessing sarcopenia status is crucial for evaluating older adults’ understanding of this condition.

This study thus aimed to examine sarcopenia knowledge, its influencing factors, and the needs for health education on sarcopenia in older Chinese community-dwelling older adults. Although logistic regression highlights the main effects of influencing factors, it does not deal well with interactions or provide good decision recommendations [12]. In contrast, decision tree models can summarize classification rules by systematically learning the attribute features of existing data, and thereby eliminating collinearity among variables [13]. The combined use of decision tree and logistic regression models can enhance analytical performance. In this study, we employed both of these methods simultaneously to analyze the factors influencing the knowledge level of sarcopenia among the older adults. The results of this study should provide a reference for improving the knowledge of sarcopenia among older adults and thereby, improving their quality of life.

## Methods

### Study design

This was a cross-sectional study that used convenience sampling to recruit 569 participants who participated in physical examination from 5 community health service center in Wuhan, China, from December 2023 to April 2024. The inclusion criteria were those who aged ≥ 60 years and were able to communicate with researchers without impediment. Older adults were excluded if they 1) had critical conditions or were in a state of end-stage organ failure, 2) had received surgery to the legs, feet, or hands in the previous 3 months, or complete disability [14], or 3) had severe cognitive dysfunction or severe visual or hearing impairments that limited cooperation.

The study was approved by the Biomedical Ethics Committee of Wuhan University on May 8, 2023, No.WHU-LFMD-IRB2023017, and all participants provided written informed consent prior to the study.

### Data collection

Data collection was based on face-to-face interviews with participants by two trained nursing graduate students and the principal investigator. The interviewers asked the questions, recorded the answers, and explained the questions that the participants could not understand. Participants were not allowed to be given any prompts regarding questions or answers throughout the data collection procedure. The average time for researchers to complete the questionnaires and scales was 15 minutes. A data entry specialist cross-checked the data entered into the computer system that was subsequently used for analysis.

### Measurement

The questionnaire consisted of part I on sociodemographic, lifestyle, and health status, part II on knowledge of sarcopenia, part III on current and expected channels to receiving sarcopenia information, and part Ⅳ on sarcopenia status.

#### Demographic questionnaire.

The researchers designed a general data questionnaire based on the purpose of the study and a literature review. The sociodemographic characteristics of the participants included age, gender, co-residence, marital status, education level, monthly income, occupation, lifestyle, and health status. Lifestyle factors were used to assess current smoking status, alcohol consumption, engagement in regular physical activity (including exercise modalities), and participation in social activities.

#### Knowledge of sarcopenia.

The knowledge levels of sarcopenia among older adults were assessed using the Sarcopenia Knowledge Awareness Survey Scale, developed by Chinese scholar Li [15] in 2023 (S1 Table). This scale consists of 10 questions to test three dimensions of sarcopenia knowledge: symptoms, risk factors, and management strategies. Responses were scored with one point allocated for a “yes” response and zero points for “do not know” or “no” responses. The total score ranged from 0 to 10, with a higher score indicating better sarcopenia knowledge. The Cronbach’s α coefficient for the scale was 0.82, and the split-half reliability was 0.86. Overall, the scale demonstrated satisfactory reliability and validity and was deemed appropriate for assessing sarcopenia knowledge in older community-dwelling adults [15]. The scale also demonstrated good internal consistency (Cronbach’s α = 0.801) in this study. Participants were classified as having an adequate or inadequate level of knowledge based on the number of correct responses. An adequate level of knowledge required at least 60% of the responses to be correct (≥6 points). If the number of correct responses was less than 60% (<6 points), the participant was classified as having inadequate knowledge [16].

#### Health education needs.

Health education needs were tested using three items: *“Do you need information about the prevention and treatment of sarcopenia? “, “Which channels do you mainly obtain knowledge about sarcopenia? “, and “In which channels would you most like to gain knowledge about sarcopenia? “.*

#### Assessment of sarcopenia.

The SARC-CalF scale was developed as a sarcopenia screening tool by Barbosa-Silva et al. [17] by combining the SARC-F scale and calf circumference (S2 Table). SARC-F scale examines five domains: (1) strength, (2) walking assistance, (3) rising from a chair, (4) climbing stairs, and (5) falls, with scores ranging between 0 and 2 [18]. The SARC-CalF is composed of 5 items from SARC-F and one additional item of calf circumference (CC). The first five items are scored as in the standard SARC-F. According to AWGS-2019, CC cut-offs were set at 34 cm for men and 33 cm for women. Scores of only 0 or 10 were assigned if CC was above or below the cut-off, respectively. A total score of ≥11 points indicated a positive screening result for sarcopenia [8]. The participants’ calf circumference was measured in a sitting position. Calf circumference was measured by trained researchers using an inelastic tape measure at the thickest part of the calf on the non-dominant side.

### Statistical analysis

All data were analyzed using IBM SPSS Statistics for Windows, version 27.0 (IBM Corp., Armonk, NY, USA). Continuous variables were checked for normality and expressed as mean (standard deviation [SD]) if normally distributed and as median (interquartile range [IQR]) if skewed. Categorical variables were expressed as number (n) and percentage (%). The chi-square test was used for bivariate analysis between characteristics of the study population and sarcopenia knowledge levels. To leverage the synergistic advantages of combining logistic regression and decision tree algorithms, all variables that were statistically significant in the bivariate analysis were included in both models. Two sided P < 0.05 was assumed to indicate a statistically significant test result for all tests.

We applied tree pruning to the decision tree algorithm to prevent overfitting of the CART prediction model and to ensure proper confirmation of the final splits. The growth depth of the model was set to have three layers. The minimum numbers of samples for parents’ and children’s nodes in the abort rule were set at 80 and 10, respectively, And the data set was split into cases for training and verification in a ratio of 6:4 Performance of the logistic regression and decision tree classifiers was measured in terms of the area under the curve (AUC) of the receiver operating characteristic (ROC) curves.

## Results

### Sociodemographic characteristics of the participants

The characteristics of the study population are presented in Table 1. A total of 569 participants were included in this study, including 223 males (39.2%) and 346 females (60.8%), with an average age of 74.48 years (SD 7.98). More than half of them were young older adults (aged <75 years) (55.5%), lived with a spouse (52.5%),were married (68.5%), had completed junior high school education or higher (65.2%), or had a monthly income < 4000 yuan (55.6%), and just under half had a BMI between 18.5–23.9 kg/m2 (47.1%). Most participants took <3 medications daily (80.3%), had 1–2 chronic diseases concurrently (65.6%), engaged in social activities regularly(72.8%), did not smoke (88.0%), and did not drink(86.8%).

**Table 1:** Characteristics of the study population by sarcopenia knowledge level.

Variable	N (%)	Insufficient Knowledge N (%)	Adequate knowledge N (%)	*χ* ^*2*^	*p*
**Sex**				0.442	0.506
Male	223 (39.2)	109 (48.9)	114 (51.1)		
Female	346 (60.8)	179 (51.7)	167 (48.3)		
**Age**				32.806	<0.001
<75	316(55.5)	126 (39.9)	190 (60.1)		
>=75	253(44.5)	162 (64.0)	91 (36.0)		
**Co-residence**				124.922	<0.001
Alone	131(23.0)	95 (72.5)	36 (27.5		
With child(ren)	151(26.5)	119 (77.9)	32 (21.2)		
With spouse	287 (50.4)	74 (25.8)	213 (74.2)		
**Marital status**				0.188	0.665
Having spouse	390 (68.5)	195 (50.0)	195 (50.0)		
No spouse	179 (31.5)	93 (52.0)	86 (48.0)		
**Occupation**				13.986	0.051
Farmer	147 (25.8)	92 (62.6)	55 (37.4)		
Factory worker	127 (22.3)	55 (43.3)	72 (56.7)		
Business staff	97 (17.0)	51 (52.6)	46 (47.4)		
Manager/Administrator	34 (6.0)	16 (47.1)	18 (52.9)		
Service staff/Sales	31 (5.4)	14 (45.2)	17 (54.8)		
Self-employed worker	29 (5.1)	13 (44.8)	16 (55.2)		
Professional and technical	83 (14.6)	36 (43.4)	47 (56.6)		
Others	21 (3.7)	11 (52.4)	10 (47.6)		
**Education level**				57.766	<0.001
Illiterate	83 (14.6)	69 (83.1)	14 (16.9)		
Primary school	115 (20.2)	68 (59.1)	47 (40.9)		
Middle school	154 (27.1)	73 (47.4)	81 (52.6)		
High school or above	217 (38.1)	78 (35.9)	139 (64.1)		
**Monthly income**				7.837	0.020
<2000RMB	122 (21.4)	71 (58.2)	51 (41.8)		
2000-4000RMB	194 (34.1)	105 (54.1)	89 (45.9)		
>4000RMB	253 (44.5)	112 (44.3)	141 (55.7)		
**Smoking**				1.304	0.253
Yes	68 (12.0)	30 (44.1)	38 (55.9)		
No	501 (88.0)	258 (51.5)	243 (48.5)		
**Drinking**				0.057	0.812
Yes	75 (13.2)	37 (49.3)	38 (50.7)		
No	494 (86.8)	251 (50.8)	243 (49.2)		
**Social activities**				21.374	<0.001
No	155 (27.2)	103 (66.5)	52 (33.5)		
Yes	414 (72.8)	185 (44.7)	229 (55.3)		
**Body mass index**				5.732	0.125
<18.5	22 (3.9)	15 (68.2)	7 (31.8)		
18.5-23.9	268 (47.1)	130 (48.5)	138 (51.5)		
24-27.9	241 (42.4)	119 (49.4)	122 (50.6)		
>=28	38 (6.7)	24 (63.2)	14 (36.8)		
**N of medications/day**				0.827	0.363
<3	457(80.3)	227 (49.7)	230 (50.3)		
>=3	112 (19.7)	61 (54.5)	51 (45.5)		
sarcopenia status				3.919	0.048
Non-sarcopenia	459 (80.7)	223 (48.6)	236 (51.4)		
sarcopenia	110 (19.3)	65 (59.1)	45 (40.9)		
**N of chronic diseases**				20.552	<0.001
0	113 (19.9)	65 (57.5)	48 (42.5)		
1-2	373 (65.6)	165 (44.2)	208 (55.8)		
>=3	83 (14.6)	58 (69.9)	25 (30.1)		

### Participants’ self-reported knowledge of sarcopenia

In our survey, we found that the participants knew very little about sarcopenia. Only 101 participants (17.8%) responded “yes” to the subjective question, “*Do you know about sarcopenia?”*, but 28.8% of participants noticed substantial muscle loss in certain elderly individuals (Table 2).

**Table 2:** Participants’ responses to subjective questions on sarcopenia.

Item	N (%)
**Do you know about sarcopenia?**	
Yes	101 (17.8)
No	468 (82.2)
**Have you observed the occurrence of substantial muscle loss in certain elderly individuals?**	
Yes	164 (28.8)
No	405 (71.2)

### Participants’ levels of sarcopenia knowledge

In the objective part of sarcopenia knowledge (Table 3), the mean score was 4.77, with a median score of 5 (1, 8) points. Among the three dimensions of sarcopenia knowledge, the questions regarding “management strategy” garnered the lowest percentage of correct answers (31.3%), followed by risk factors (48.9%). Moreover, the accuracy rate of symptom-related knowledge scores was only 54.7%. Regarding the risk factors of sarcopenia, the majority of participants attributed sarcopenia to advanced age (59.2%) and chronic bed rest (58.2%). A small number of participants (28.8%) indicated that having chronic diseases was a risk factor for sarcopenia. Furthermore, in terms of management strategy, only a small percentage of participants were aware that appropriate resistance exercise (27.1%) and adequate protein intake (35.5%) could prevent or relieve sarcopenia. (Table 3).

**Table 3:** Participants’ answers to objective questions on sarcopenia.

Item	Yes N (%)	Do not know N (%)	No N (%)
**Symptom**	569 (54.7)	569 (41.6)	569 (3.7)
Is it possible for patients with sarcopenia to have slender or weak limbs?	299 (52.5)	251 (44.1)	19 (3.3)
Is it possible for patients with sarcopenia to have decreased grip strength?	286 (50.3)	260 (45.7)	23 (4.0)
Is it possible for patients with sarcopenia to walk at a slower pace?	333 (58.5)	212 (37.3)	24 (4.2)
Is it possible for patients with sarcopenia to have decreased exercise capacity?	327 (57.5)	224 (39.4)	18 (3.1)
**Risk Factor**	569 (48.9)	569 (45.8)	569 (5.3)
Are older adults more likely to suffer from sarcopenia with aging?	337 (59.2)	199 (35.0)	33 (5.8)
Are chronically bedridden older adults more likely to suffer from sarcopenia?	331 (58.2)	222 (39.0)	16 (2.8)
Are old adults with chronic diseases (e.g., diabetes, hyperlipidemia) more likely to suffer from sarcopenia?	164 (28.8)	356 (62.6)	49 (8.6)
Are malnourished older adults more likely to suffer from sarcopenia?	282 (49.6)	266 (46.7)	21 (3.7)
**Management Strategy**	569 (31.3)	569 (64.0)	569 (4.7)
Can appropriate resistance training (e.g., elastic bands, dumbbells, sandbags) prevent or relieve sarcopenia?	154 (27.1)	395 (69.4)	20 (3.5)
Can adequate protein intake prevent or relieve sarcopenia?	202 (35.5)	333 (58.5)	34 (6.0)

### Comparison of participants’ self-assessed versus measured sarcopenia

The self-assessed sarcopenia of participants did not match the results measured using SARC-CalF. There were 29.0% of participants who could not accurately identify their sarcopenia status. The majority of participants responded that they had no experienced a significant reduction in muscle mass (77.3%). Among participants who screened positive for sarcopenia, only 33.6% could accurately self-identify their muscle status (Table 4).

**Table 4:** Comparison of participants’ self-assessed versus measured sarcopenia.

Measured sarcopenia status	Self-assessed sarcopenia status		
	Yes	No	Correct (%)
No	92	367	80.0
Yes	37	73	33.6
Overall percentage (%)	22.7	77.3	71.0

### Health education needs of participants

**Fig 1:**
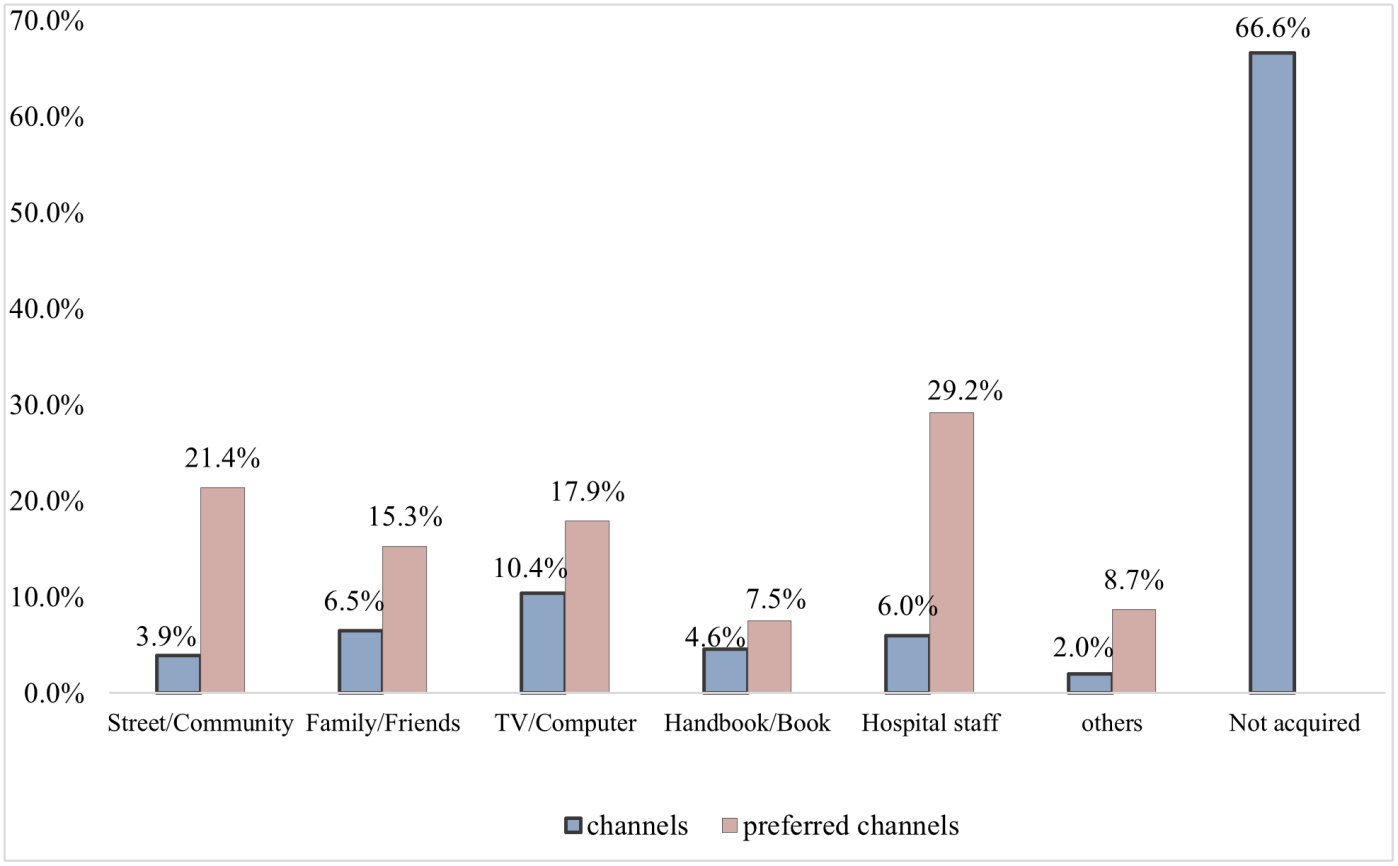
Participants’ current and preferred channels for acquiring sarcopenia knowledge.

In this survey, most of the participants (66.6%) had never acquired knowledge about sarcopenia, but 72.4% of the participants wanted to acquire knowledge about sarcopenia. Among their current channels for obtaining sarcopenia knowledge, TV networks (10.4%) and family and friends (6.5%) were the most commonly mentioned. However, a plurality of participants preferred to receive sarcopenia knowledge through healthcare professionals’ introduction (29.2%), and community events (21.4%), followed by television networks (17.9%). The proportion of those who wanted to obtain relevant knowledge through handbooks (7.5%) was significantly lower than the above three channels (Fig 1).

### Logistic regression on factors associated with sarcopenia knowledge

In our bivariate analysis, participant’ age, monthly income, co-residence, chronic diseases, education level, sarcopenia status and social activities were found to be significantly associated with their sarcopenia knowledge (Table 1).These factors were further assessed in a binary logistic regression using a stepwise method. The results of this analysis showed that age, co-residence, educational level, social activities, and chronic diseases were all significant factors associated with knowledge about sarcopenia (Table 5).

**Table 5:** Logistic regression of factors associated with sarcopenia knowledge levels among the participants.

Variable		Beta	Wald	*P*	OR (95%CI)
Age	<75	Reference			
	>=75	−0.759	11.741	<0.001	0.468 (0.303-0.723)
Monthly income	<2000	Reference			
	2000-4000	−0.149	0.242	0.623	0.862 (0.476-1.560)
	>4000	−0.076	0.051	0.821	0.926 (0.478-1.796)
Social activities	No	Reference			
	Yes	1.037	17.691	<0.001	2.821(1.740-4.573)
Co-residence	Alone	Reference			
	With child(ren)	−0.330	1.119	0.290	0.719 (0.390-1.325)
	With spouse	1.840	46.977	<0.001	6.296 (3.720-10.655)
sarcopenia status	Non-sarcopenia	Reference			
	sarcopenia	0.176	0.409	0.522	1.192 (0.695-2.045)
Education level	Illiterate	Reference			
	Primary school	0.763	3.482	0.062	2.145 (0.962-4.781)
	Middle school	1.238	9.540	0.002	3.447 (1.572-7.560)
	High school or above	1.618	14.972	<0.001	5.044 (2.222-11.448)
N of chronic diseases	0	Reference			
	1-2	0.810	9.338	0.002	2.248 (1.337-3.780)
	>=3	0.103	0.077	0.781	1.109 (0.536-2.294)

### Analysis of differences in logistic regression models by age subgroup

We further analyzed different age subgroups (Table 6). Co-residence, social activities and education level emerged as independent factors across all age groups. Among those younger than 75 years, participants with 1–2 chronic diseases showed higher awareness of sarcopenia.

**Table 6:** Analysis of differences in logistic regression models by age subgroup.

Variable	<75		>=75	
	OR (95%CI)	*p*	OR (95%CI)	*p*
**Monthly income (reference:** < 2000)				
2000-4000	0.939 (0.433-2.034)	0.873	0.777 (0.295-2.045)	0.610
>4000	1.050 (0.433-2.547)	0.914	0.748 (0.266-2.100)	0.581
**Social activities (reference:** No)				
Yes	3.194 (1.624-6.283)	0.001	2.341 (1.143-4.795)	0.020
**Co-residence (reference:** Alone)				
With child (ren)	1.247 (0.498-3.121)	0.638	0.469 (0.188-1.173)	0.106
With spouse	11.701 (4.929-27.778)	<0.001	4.280 (2.137-8.574)	<0.001
**sarcopenia status (reference:** Non-sarcopenia)				
sarcopenia	1.267 (0.544-2.950)	0.583	1.078 (0.529-2.196)	0.836
**Education level (reference:** Illiterate)				
Primary school	3.053 (0.983-9.488)	0.054	1.417 (0.440-4.558)	0.559
Middle school	4.091 (1.356-12.346)	0.012	2.627 (0.853-8.090)	0.092
High school or above	5.818 (1.838-18.414)	0.003	4.131(1.263-13.506)	0.019
**N of chronic diseases (reference:** 0)				
1-2	2.578 (1.309-5.077)	0.006	2.006 (0.825-4.876)	0.125
>=3	0.644 (0.222-1.872)	0.419	1.548 (0.525-4.566)	0.428

### Decision tree model of influencing factors for sarcopenia knowledge

**Fig 2:**
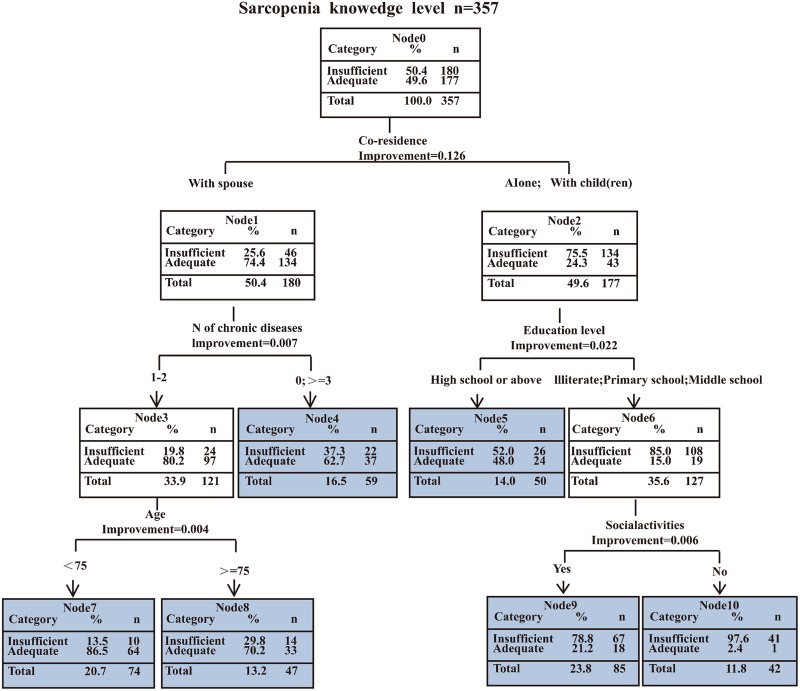
Decision tree model of influencing factors for participants’ sarcopenia knowledge.

Fig 2 presents the optimal decision tree for older adults’ sarcopenia knowledge levels. Residing with a spouse had the most substantial effect on sarcopenia knowledge among all explanatory variables, followed by education level, social activities, age, and chronic diseases (S3 Table).The group with the highest proportion of low sarcopenia knowledge level consisted of those who lived alone or with children, never attended school or completed primary or middle school only, and never engaged in any social activities (97.6%). The group with the highest proportion of high sarcopenia knowledge consisted of those individuals who lived with spouse, had 1–2 chronic diseases concurrently, and whose age was below 75 years (86.5%). The training samples of the decision tree model had an accuracy of 75.1% (sensitivity 74.4% and specificity 75.7%), and the test samples showed 75.0% accuracy (sensitivity74.1% and specificity 76.0%).

**Fig 3:**
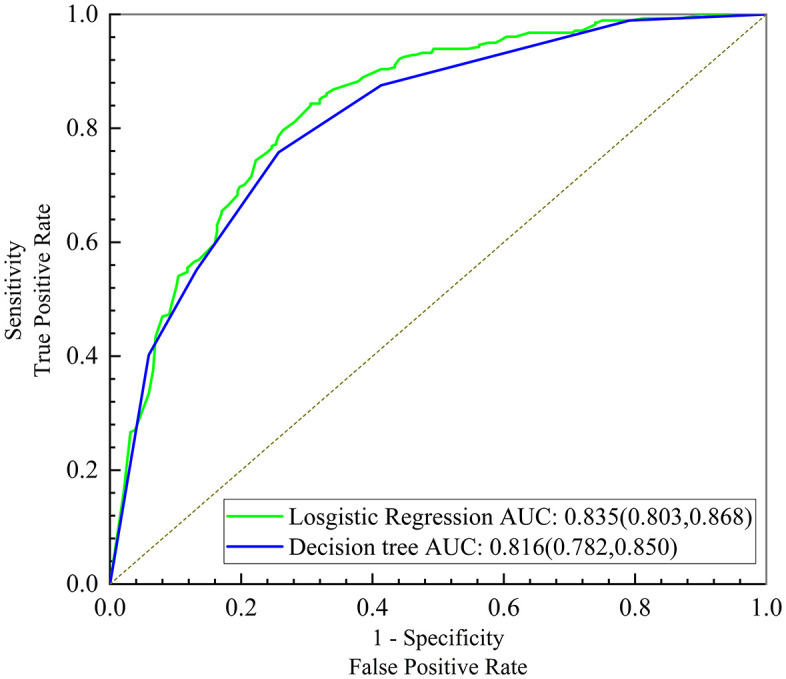
ROC curves of the logistic regression and decision tree models on sarcopenia knowledge.

The AUC of the logistic regression and decision tree models to predict sarcopenia knowledge levels were 0.835 (95% CI: 0.803, 0.868) and 0.816 (95% CI: 0.782, 0.850) respectively, and their difference was statistically significant (95% CI: 0.001, 0.037, P = 0.036) (Fig 3).

## Discussion

This study contributes to the limited quantitative literature on sarcopenia knowledge among older Chinese community-dwelling adults, with a particular focus on associated sociodemographic and behavioral determinants. Our results revealed that the older adults in Chinese communities have little knowledge about sarcopenia, particularly in the dimensions of management strategy and risk factors. Furthermore, there was a significant discrepancy between the participants’ subjective assessments of their sarcopenia condition and our objective measurements (Table 4). There was also a pronounced demand for health education about sarcopenia among the participants. Logistic regression analysis showed that that age, co-residence, educational level, social activities, and chronic diseases were all significant factors associated with knowledge about sarcopenia. In addition, the decision tree model indicated that individuals who lived alone or with children, possessed a lower level of education, and were not engaged in social activities tended to have the lowest sarcopenia knowledge levels.

### Low levels of knowledge about sarcopenia

Our study revealed that 54.7% of older adults could identify sarcopenia symptoms, consistent with the 56.9% reported in the Malaysian study [7]. However, this apparent recognition warrants cautious interpretation, as older adults frequently conflate sarcopenia symptoms with normal aging-related physiological changes, such as the gradual decline in skeletal muscle mass and strength [7]. The relatively short research and clinical history of sarcopenia has led to a very low level of public awareness and confusion with the symptoms of normal aging [8,19].

In terms of perception of the risk factors for sarcopenia, nearly half (48.9%) of the participants did not know what the risk factors were. In particular, up to 71.8% of the participants were unaware of the negative effects of chronic disease on sarcopenia. Chronic diseases can increase the energy consumption of the body, accelerate metabolic activity, and cause chronic inflammation. Chronic inflammation is a persistent, low-grade inflammatory response that can negatively affect multiple bodily systems, including muscle tissue. When chronic inflammation persists, it interferes with the balance between anabolism and catabolism of muscle, and this imbalance leads to progressive loss of muscle tissue, which triggers sarcopenia [20]. In addition, chronic inflammation may also be accompanied by neurodegenerative changes, which further aggravate the development of sarcopenia. Intrinsic molecular mechanisms such as aging and malnutrition may also be related to the occurrence and development of sarcopenia, which further aggravate the impact of chronic diseases on individual health by affecting muscle mass and function [21].Therefore, policy makers and healthcare providers may want to establish a health education system, with the goal of popularizing health knowledge, and guiding the older adults toward more beneficial health choices. Furthermore, personalized health intervention should be provided for the elderly, and risk assessment and intervention guidance given for those at higher risk of sarcopenia due to chronic disease.

In terms of awareness of preventive measures for sarcopenia, only 27.1% of participants were aware that resistance training can help prevent sarcopenia, and just 35.5% knew that adequate protein supplementation could serve the same purpose. This floor effect reflects a significant lack of awareness among older adults regarding the importance of resistance exercise and dietary protein intake. Low awareness among older adults regarding the benefits of resistance training for sarcopenia improvement may be associated with insufficient knowledge about resistance training, cultural preferences, and fear of injury. The results of Chun Qin Y et al. [22]suggest that majority of the older adults in China do not know what resistance training is, and some may be reluctant to engage in such activities due to the fear of falling [23]. Such knowledge limitations manifest in behavioral patterns. In a cross-sectional survey of 113 elderly people conducted by Sun Yujia et al.[24] across various locations in Henan Province, the majority of the elderly preferred walking as their primary form of physical exercise (82.5%), followed by sports dancing (square dancing) (32%). Furthermore, in a study on the preference for fitness activities among Chinese middle-aged and elderly people, Chen Yong et al.[25] pointed out that many individuals believe that the time and effort invested in fitness activities are not proportional to the actual benefits obtained due to the lack of professional guidance. Therefore, these individuals tend to choose gentle and low-intensity fitness activities, such as Tai Chi, jogging, Qigong, swimming, and square dancing. The above studies show that Chinese older adults have a low cognition of resistance training on the whole. Unfortunately, nutritional knowledge gaps parallel these exercise-related deficiencies. For example, Spronk et al.’s [26] study showed that less nutrition-related knowledge is associated with worse eating behaviors. Additionally, Marije et al. found that low education levels and lack of social support are independently associated with low protein intake [27]. Therefore, the low awareness among participants in this study that protein intake can prevent sarcopenia may be due to a lack of awareness of the need for dietary protein in older adults more generally. Integrating both domains, AWGS-2019 notes that lifestyle interventions, especially exercise and nutritional supplements, have potential long-term benefits for sarcopenia. Therefore, strides may be made toward optimal health by guiding the older adults to a better understanding of the benefits of resistance training and protein supplementation for delaying the occurrence and development of sarcopenia.

### Factors that influencing knowledge of sarcopenia

The results of this study showed that aging and lower education levels were risk factors for low levels of knowledge about sarcopenia among older adults. Aging is correlated with a decline in cognitive processes, especially in working memory, which affects people’s abilities to acquire and retain health-related information [28,29]. In contrast, younger seniors can participate in more social activities and have the ability to seek health-related information more actively [30,31]. Evidence suggests that people with higher levels of education have a better ability to acquire and process health-related knowledge [32,33]. In addition, educational interventions can significantly increase health knowledge among older adults [34].

Our results also highlight the fact that living alone or with children is a risk factor for a low level of knowledge of sarcopenia in older adults, and the level of knowledge of sarcopenia was higher in older adults who lived with a spouse. This suggests that the support of health information from children is limited. Studies have suggested that spouse support might exert the most significant influence on older adults’ well-being, even more so than supports from other family members or friends [35]. Older adults who live with spouses can supervise, support and encourage each other in health-related issues [36]. Spouses engage in more frequent health information exchange, shared health behaviors, and mutual emotional support in daily life [37,38]. For example, they may encourage healthy habits such as regular physical activity, which is associated with the onset of sarcopenia. Furthermore, a spouse with higher education is more likely to share knowledge about health promotion [37]. Interventions that focus on the spouse as a unit could thus enhance patients’ self-management activities [39], improve patient compliance [40] and quality of life [41], and help to maintain supportive relationships [42].

Older adults who participated in social activities also had a higher level of sarcopenia knowledge. One possible reason is that older people discuss their common health problems with each other in the course of social activities. Evidently, the health problems caused by muscle loss are common problems for them. Studies have shown that social participation even provides opportunities for socioeconomically disadvantaged groups to receive health-related information from others [43–45], resulting in increased acquisition of health-related knowledge. Interestingly, our study revealed that older adults with 1–2 chronic diseases were more knowledgeable about sarcopenia. Other studies have also found that patients with a certain chronic disease are more likely to have a higher level of health knowledge [46–48]. One possible explanation for this might be that people with 1–2 chronic diseases demonstrate increased attention to their health, which prompts them to obtain health information more actively [49]. Moreover, previous studies have indicated that it is difficult for older people with multiple chronic diseases to access and manage health information effectively due to technological, emotional, and systemic challenges [50–52]. One cross-sectional study from China showed an inverse association between nutritional health knowledge and the number of chronic diseases, especially among participants with three or more chronic diseases [53]. In our study, most older adults were unaware that chronic diseases are a risk factor for sarcopenia. Therefore, to improve the knowledge levels of sarcopenia in the older adults, it may be necessary to pay close attention to the elderly people with multiple chronic diseases.

Subgroup analyses indicated that knowledge of sarcopenia was associated with living arrangements, social activity, education level, and chronic disease status, and this consistency with the overall population analysis further confirms the robustness of the results.

Although logistic regression achieved marginally higher predictive accuracy (Fig 3), decision tree models are mostly presented in the form of tree diagrams, that are simpler to interpret and hence more convenient for clinical decision-making. The ranking of variable importance in decision trees also provides a clear priority framework for developing intervention strategies. Co-residence exerted the most significant influence on sarcopenia knowledge levels among older adults. Consequently, interventions should prioritize addressing the distinct needs associated with different living arrangements, and design targeted strategies aligned with their specific information access characteristics. The group with the highest proportion of inadequate sarcopenia knowledge consisted of those who lived alone or with children, had an education level of middle school or below, and never engaged in any social activities. Therefore, based on the results of this study, we recommended that policymakers take as a starting point in increasing the knowledge level regarding sarcopenia in older adults a concerted effort to understand the living arrangement, education, and social participation of older adults and implementing targeted health education strategies for people with different backgrounds.

### Methods to improve the sarcopenia knowledge

AWGS-2019 Guidelines [8] states that the promotion of early lifestyle interventions in primary health care centers and communities are more beneficial for improving the quality of life of older people with sarcopenia. Our study showed that the vast majority (72.4%) of participants were willing to learn about sarcopenia and preferred to obtain relevant information from medical professionals and primary health care personnel. However, studies by Yeung SSY [54], Ji M [55], and Guralnik [56] show that except in specific departments, the understanding and application of sarcopenia among medical personnel is very limited. One qualitative study of nurses in primary care services showed that nurses had little knowledge of sarcopenia [57]. In China, the tasks of community health professionals are more concentrated on chronic diseases such as hypertension and diabetes [58]. Training community healthcare workers may thus be a pivotal strategy for enhancing sarcopenia screening and education within communities. Improving health care workers’ awareness of sarcopenia and reaching a broad consensus on its diagnostic criteria, screening algorithms, treatment guidelines, prevention strategies, patient education and interdisciplinary collaboration may help to identify high risk individuals and to delivery initial health education in community settings, thereby addressing limitations in specialist resources. The Chinese government has already promulgated the Key Tasks for Deepening the Reform of the Medical and Health System [59], which directs the allocation of medical resources to be tilted to the community level, and may thusly help improve the capacity of primary medical services. Hospitals and communities should respond to the national call to actively disseminate knowledge about sarcopenia. While possessing health knowledge is crucial, it does not automatically translate into healthy behaviors. Thus, future health education and community-based interventions should extend beyond knowledge dissemination to improve functional health literacy among older adults. Enhancing these skills will empower them to access, comprehend, and apply health information for informed decision-making [60], thereby facilitating the adoption of healthier behaviors.

### Strengths and limitations of the Study

This study contributes to the limited quantitative literature on sarcopenia knowledge among older Chinese community-dwelling adults. It used both logistic regression and a decision tree model to explore factors that influence this knowledge level, and the two methods complement each other. Moreover, our study not only identified many modifiable risk factors that affect sarcopenia knowledge, but also investigated several channels by which older adults can obtain sarcopenia-related health information. Our results provided a basis for tailored sarcopenia education interventions for older adults in Chinese communities.

There are several limitations to our study. First, as a cross-sectional study, it only reflects the current knowledge level of the population surveyed, and cannot assess dynamic changes in knowledge levels or whether knowledge has been translated into behaviors. Second, participants were recruited only from Wuhan city, reducing the generalizability of our results. Third, sarcopenia status was assessed using the SARC-CalF rather than a full clinical assessment. Future research should aim to validate these findings in cohorts with rigorously confirmed sarcopenia diagnoses using objective measures of muscle mass and function. Finally, self-reported knowledge and behaviors may be subject to recall bias or social desirability bias, especially in older adults. Future randomized controlled trials or longitudinal studies are needed to examine the effectiveness of sarcopenia health education on knowledge, and answer the question of whether health knowledge is translated into health behaviors, especially for those at high risk of sarcopenia

## Conclusion

Older Chinese community-dwelling adults were found to have a low level of knowledge about sarcopenia, especially those who lived alone or with children, those with low education, never engaged in any social activities, and those aged over 75 years. We suggest that further educational programs and tailored intervention strategies should be implemented to improve the knowledge levels of sarcopenia in this population.

## Supporting information

S1 Table Sarcopenia knowledge assessment scale. (DOCX)

S2 Table The SARC-CalF scales. (DOCX)

S3 Table Variable importance in the decision tree model. (DOCX)
