# Supplementary material for: The knowledge level and influencing factors of sarcopenia among Chinese community-dwelling older adults
Source: PLoS One. 2025 Oct 16;20(10):e0333557. doi: 10.1371/journal.pone.0333557 (PMC12530540; doi:10.1371/journal.pone.0333557)
Supplement: S1 Table — (DOCX) [file pone.0333557.s001.docx]

**S1 Table** Sarcopenia Knowledge Assessment Scale

| Dimension | Item | Options(score) | | |
| --- | --- | --- | --- | --- |
| Symptom | 1. Is it possible for patients with sarcopenia to have slender or weak limbs? | Yes=1 | Do not know=0 | No=0 |
|  | 2. Is it possible for patients with sarcopenia to have decreased grip strength? | Yes=1 | Do not know=0 | No=0 |
|  | 3. Is it possible for patients with sarcopenia to walk at a slower pace? | Yes=1 | Do not know=0 | No=0 |
|  | 4. Is it possible for patients with sarcopenia to have decreased exercise capacity? | Yes=1 | Do not know=0 | No=0 |
| Risk Factor | 5. Are older adults more likely to suffer from sarcopenia with aging? | Yes=1 | Do not know=0 | No=0 |
|  | 6. Are chronically bedridden older adults more likely to suffer from sarcopenia? | Yes=1 | Do not know=0 | No=0 |
|  | 7. Are old adults with chronic diseases ( e.g., diabetes, hyperlipidemia) more likely to suffer from sarcopenia? | Yes=1 | Do not know=0 | No=0 |
|  | 8. Are malnourished older adults more likely to suffer from sarcopenia? | Yes=1 | Do not know=0 | No=0 |
| Management Strategy | 9. Can appropriate resistance training (e.g., elastic bands, dumbbells, sandbags)prevent or relieve sarcopenia? | Yes=1 | Do not know=0 | No=0 |
|  | 10. Can adequate protein intake prevent or relieve sarcopenia? | Yes=1 | Do not know=0 | No=0 |
