# Supplementary material for: The knowledge level and influencing factors of sarcopenia among Chinese community-dwelling older adults
Source: PLoS One. 2025 Oct 16;20(10):e0333557. doi: 10.1371/journal.pone.0333557 (PMC12530540; doi:10.1371/journal.pone.0333557)
Supplement: S2 Table — (DOCX) [file pone.0333557.s002.docx]

**S2 Table** The SARC-CalF Scales

| Components | Questions | SARC-CalF Score |
| --- | --- | --- |
| Strength | Did you experience any difficulty in lifting or carrying 10 pounds? | None = 0 Some = 1 Great difficulty or unable to lift = 2 |
| Assistance in walking | Did you experience any difficulty in walking across a room? | None = 0 Some = 1 Great difficulty, use aids, or unable to walk = 2 |
| Rising from a chair | Did you experience any difficulty in transferring from a chair or bed? | None = 0 Some = 1 Great difficulty or unable to transfer without help = 2 |
| Climbing stairs | Did you experience any difficulty in climbing a flight of 10 steps? | None = 0 Some = 1 Great difficulty or unable to climb = 2 |
| Falls | Did you experience any falls in the past year? | None = 0 1–3 falls = 1 4 or more falls = 2 |
| Calf circumference |  | Females: > 33cm = 0  ≤33cm = 10 Males: >34cm = 0 ≤34cm =10 |
