# Supplementary material for: The knowledge level and influencing factors of sarcopenia among Chinese community-dwelling older adults
Source: PLoS One. 2025 Oct 16;20(10):e0333557. doi: 10.1371/journal.pone.0333557 (PMC12530540; doi:10.1371/journal.pone.0333557)
Supplement: S3 Table — (DOCX) [file pone.0333557.s003.docx]

**S3** **Table** Variable importance in the decision tree model

| Variable | importance | Normalized Importance% |
| --- | --- | --- |
| Co-residence | 0.126 | 100% |
| Education level | 0.063 | 50.4% |
| Social activities | 0.037 | 29.2% |
| Age | 0.028 | 21.9% |
| N of chronic diseases | 0.014 | 10.9% |
